# Complete Genome Sequence of a *Mycobacterium tuberculosis* Strain Belonging to the East African-Indian Family in the Indo-Oceanic Lineage, Isolated in Hanoi, Vietnam

**DOI:** 10.1128/genomeA.00509-17

**Published:** 2017-06-15

**Authors:** Takayuki Wada, Minako Hijikata, Shinji Maeda, Nguyen Thi Le Hang, Pham Huu Thuong, Nguyen Phuong Hoang, Nguyen Van Hung, Naoto Keicho

**Affiliations:** a Department of International Health, Institute of Tropical Medicine, Nagasaki University, Nagasaki, Japan; b Department of Pathophysiology and Host Defense, Research Institute of Tuberculosis, Kiyose, Tokyo, Japan; c Hokkaido Pharmaceutical University School of Pharmacy, Sapporo, Hokkaido, Japan; d NCGM-BMH Medical Collaboration Center, Hanoi, Vietnam; e Hanoi Lung Hospital, Hanoi, Vietnam; f Department of Microbiology, Hanoi Lung Hospital, Hanoi, Vietnam; g Department of Microbiology, National Lung Hospital, Hanoi, Vietnam; h National Center for Global Health and Medicine, Tokyo, Japan

## Abstract

The East African-Indian (EAI) family of *Mycobacterium tuberculosis* is an endemic group mainly observed in Southeast Asia. Here, we report the complete genome sequence of an *M. tuberculosis* strain isolated as a member of the EAI family in Hanoi, Vietnam, a country with a high incidence of tuberculosis.

## GENOME ANNOUNCEMENT

The incidence of tuberculosis in Vietnam was estimated to be 128 per 100,000 individuals in 2015 (1). In Hanoi, the capital city of the country, a population-based study to determine the genetic background of *Mycobacterium tuberculosis* was conducted by our group from 2007 to 2009, and 465 clinical strains were isolated (2). Although Beijing family strains belonging to the East Asian lineage (lineage 2) (3, 4) were predominant (58.5%) in Hanoi, the East African-Indian (EAI) family belonging to an ancestral Indo-Oceanic lineage (lineage 1), isolated from Southeast Asian countries and other regions (5), has also been isolated (17.6%) in the same area (2). In Vietnam, the influence of EAI family strains may exceed that of Beijing family strains in rural areas, indicating that the EAI family may have been indigenous to this region before the Beijing family started to spread (6, 7).

Here, we describe the complete genome sequence of HN-024, an EAI4-VNM strain of *M. tuberculosis*, defined by *in silico* spoligotyping. This strain was isolated from a 58-year-old Vietnamese woman before initial treatment for tuberculosis in 2007 (2). The strain showed no resistance to rifampin, isoniazid, streptomycin, ethambutol, or pyrazinamide by drug susceptibility testing and a pyrazinamidase assay. A PacBio RS II instrument (Pacific Biosciences, CA) was used to determine the complete genome sequence. Long-read sequences were obtained (399.8 Mb, with 4,821 bp as the average length of read inserts) and assembled with Hierarchical Genome Assembly Process (HGAP) version 3. Consequently, a single contig over 4.4 Mb in length was assembled. The contig was polished by a mapping analysis of 300-bp paired-end reads obtained by MiSeq sequencing (Illumina, CA), using CLC Genomics Workbench (Qiagen, CA, USA). After polishing, two regions, including repetitive structures by remapping analysis, remained, and their nucleotide sequences were directly determined by Sanger sequencing for confirmation.

Finally, the complete genome sequence was 4,399,916 bp, with 65.6% G+C content. These values were similar to previously reported complete genomes, such as those of H37Rv (a reference strain, with 4,411,532 bp and 65.6% G+C content) (8) and EAI5 (a member of the EAI family, with 4,391,174 bp and 65.6% G+C content) (9). Prior to submission to DDBJ, D-FAST (10), a pipeline, including annotation by PROKKA version 1.11 (11), was applied to the genome sequence, which predicted 4,027 protein-coding regions and 52 tRNAs. To improve the compatibility and usability of the new sequence, the original annotation by D-FAST was modified to include locus tags and gene names of the H37Rv genome (GenBank accession no. AL123456.3), according to reciprocal BLASTP best hits by standalone BLAST+ (version 2.2.29) (12).

Because the genome sequence reported in this study was obtained from a typical Vietnamese EAI strain, it could serve as a reference for comparative genomics of *M. tuberculosis* and may provide clues to elucidate the history of the spread of EAI family strains in Vietnam.

### Accession number(s).

This whole-genome sequencing study has been deposited at DDBJ/ENA/GenBank under the accession number AP018033. The version described in this paper is the first version.
